# Supplementary figures and images for: Nintedanib ameliorates experimental pulmonary arterial hypertension via inhibition of endothelial mesenchymal transition and smooth muscle cell proliferation
Source: PLoS One. 2019 Jul 24;14(7):e0214697. doi: 10.1371/journal.pone.0214697 (PMC6656344; doi:10.1371/journal.pone.0214697)

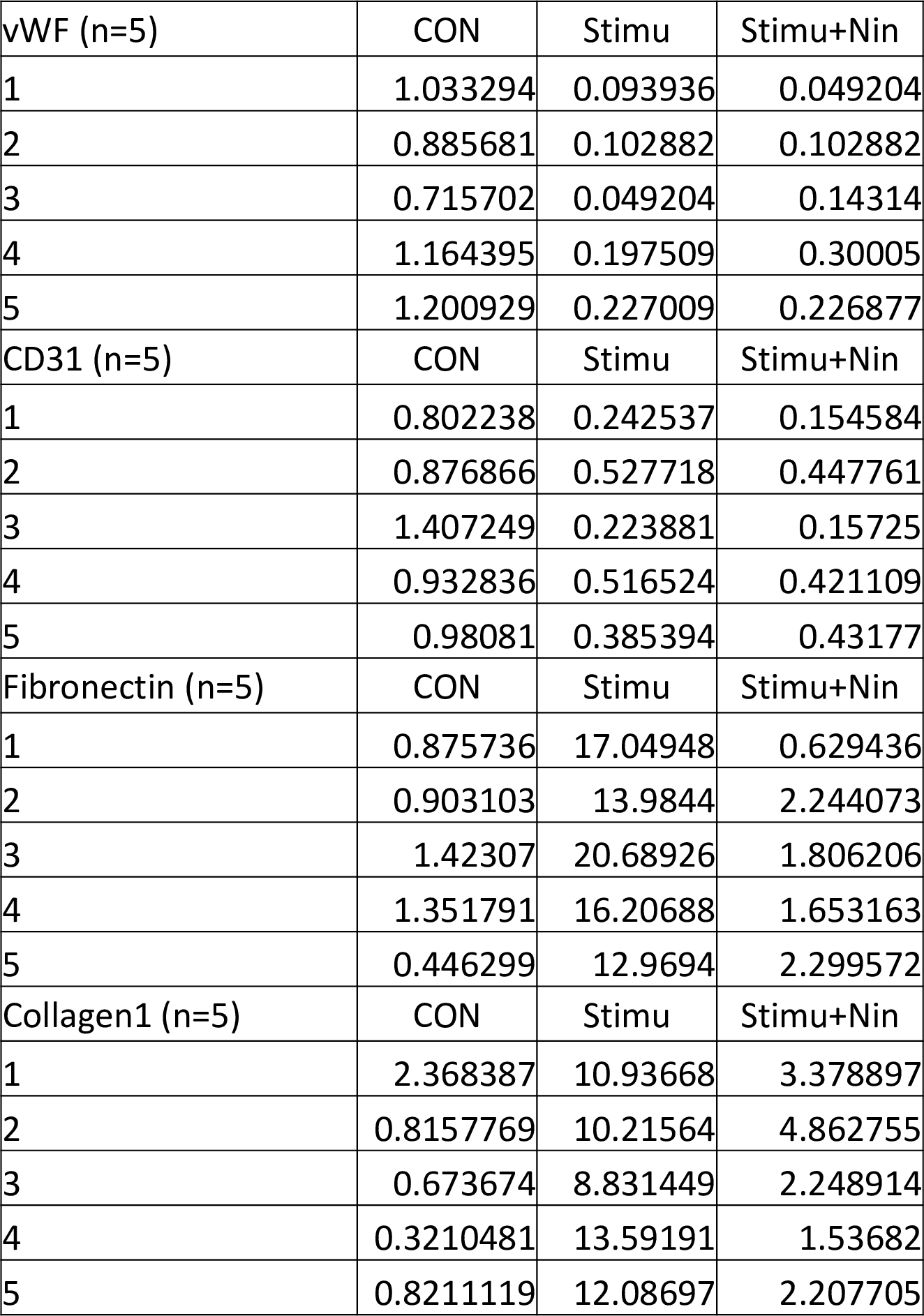

Supplement: S1 Table — WF: von Willebrand factor. Stimu: stimulation with TGF-β2, TNF-α, and IL-1β. Nin: nintedanib. (TIF) [file pone.0214697.s001.tif]

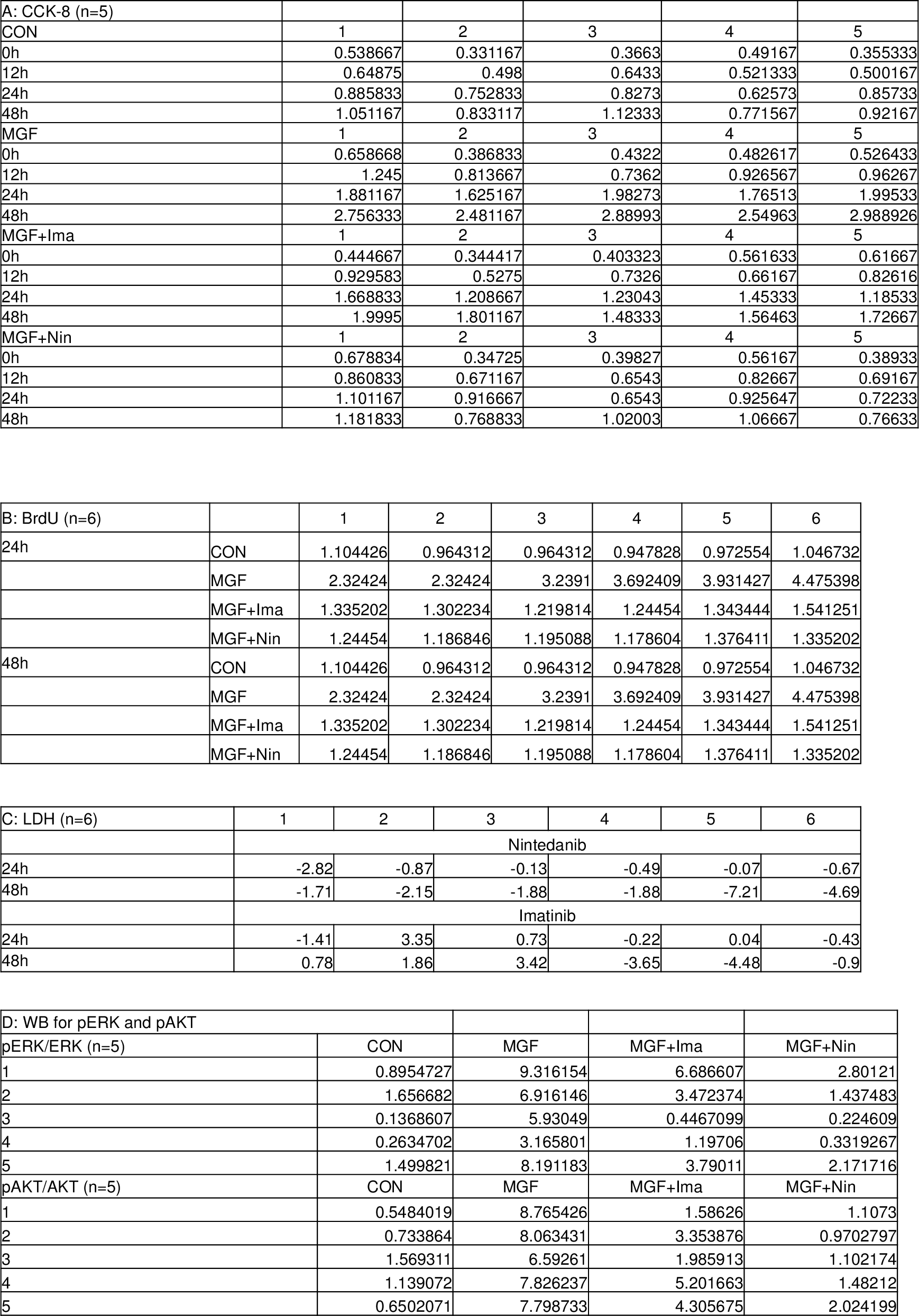

Supplement: S2 Table — Individual values for pERK/ERK and pAKT/AKT by densitometric analysis of western blotting are also in table. Ima: imatinib. Nin: nintedanib. CON: control. MGF: multiple growth factors. (TIF) [file pone.0214697.s002.tif]

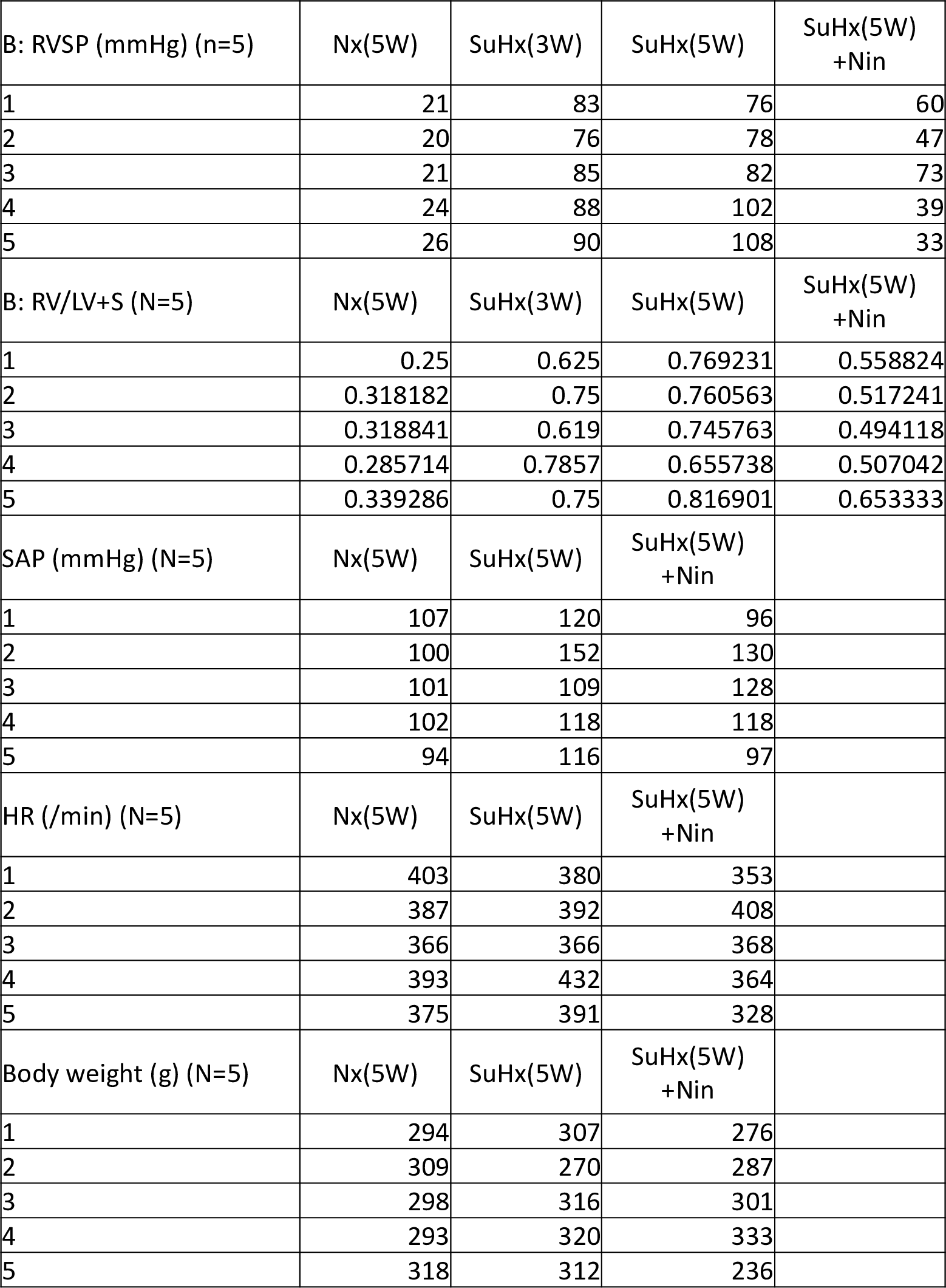

Supplement: S3 Table — RVSP: right ventricle systolic pressure. RV/LV+S: right ventricle weight / (left ventricle and septal weight) ratio. SAP: systemic systolic arterial pressure. HR: heart rate. Nx: control rat with normoxic condition. SuHx: single injection of Sugen 5416 with chronic hypoxic exposure. Nin: nintedanib. W: week. (TIF) [file pone.0214697.s003.tif]

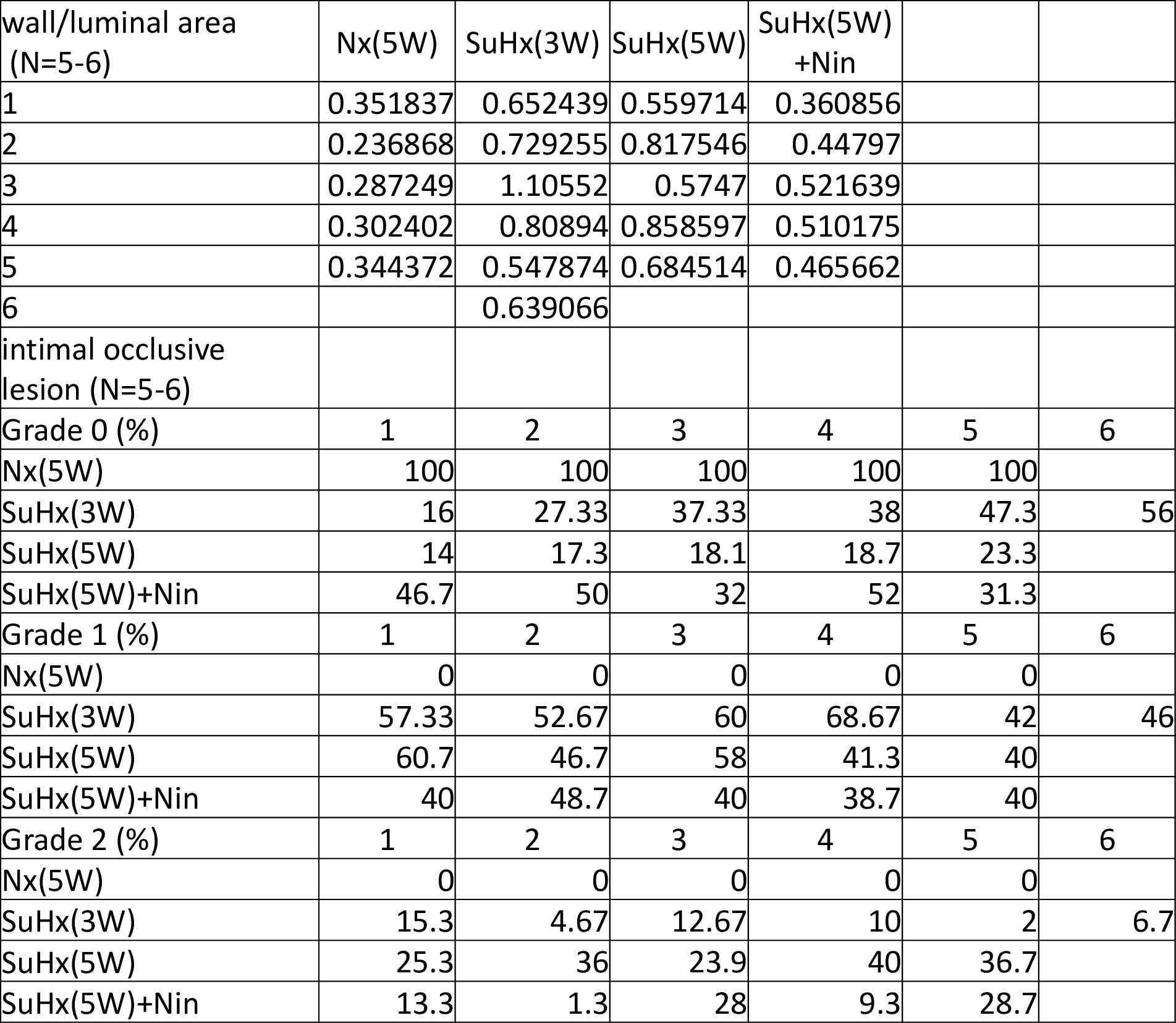

Supplement: S4 Table — Nx: control rat with normoxic condition. SuHx: single injection of Sugen 5416 with chronic hypoxic exposure. Nin: nintedanib. W: week. (TIF) [file pone.0214697.s004.tif]

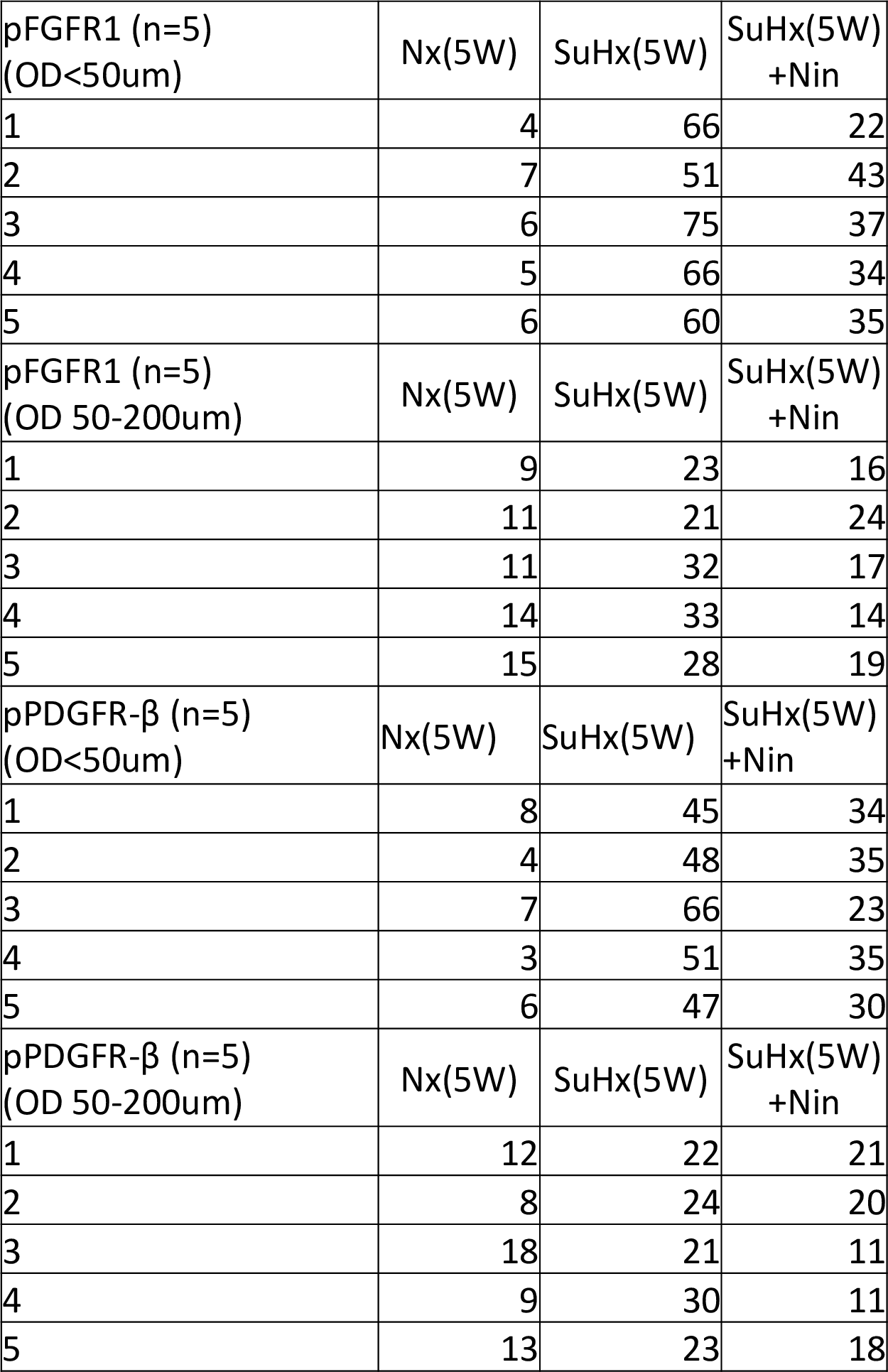

Supplement: S5 Table — Nx: control rat with normoxic condition. SuHx: single injection of Sugen 5416 with chronic hypoxic exposure. Nin: nintedanib. W: week. OD: outer diameter. (TIF) [file pone.0214697.s005.tif]

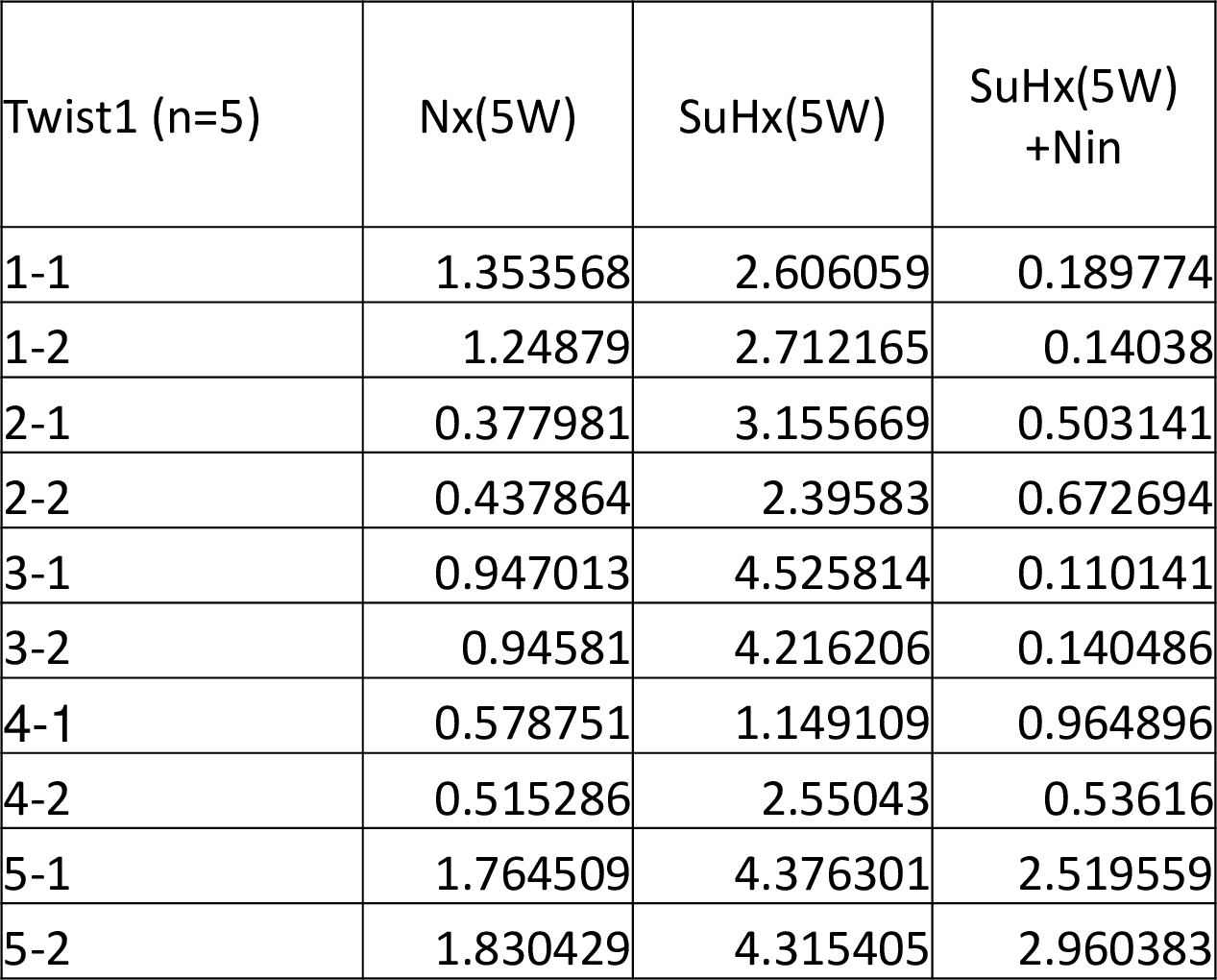

Supplement: S6 Table — Nx: control rat with normoxic condition. SuHx: single injection of Sugen 5416 with chronic hypoxic exposure. Nin: nintedanib. W: week. (TIF) [file pone.0214697.s006.tif]
